# A step-by-step guide to systematically identify all relevant animal studies

**DOI:** 10.1258/la.2011.011087

**Published:** 2012-01

**Authors:** Marlies Leenaars, Carlijn R Hooijmans, Nieky van Veggel, Gerben ter Riet, Mariska Leeflang, Lotty Hooft, Gert Jan van der Wilt, Alice Tillema, Merel Ritskes-Hoitinga

**Affiliations:** 1 3R Research Centre, Central Animal Laboratory, Radboud University Nijmegen Medical Centre, PO Box 9101, 6500 HB Nijmegen, The Netherlands; 2 Centre for Equine and Animal Science, Writtle College, Chelmsford, Essex CM1 3RR, UK; 3 Department of General Practice, Academic Medical Centre, University of Amsterdam, Amsterdam, The Netherlands; 4 Dutch Cochrane Centre, Amsterdam, The Netherlands; 5 Netherlands Trial Register, Amsterdam, The Netherlands; 6 Department of Epidemiology, Biostatistics, and Health Technology Assessment, Radboud University Nijmegen Medical Centre, Nijmegen, The Netherlands; 7 Medical Library, Radboud University Nijmegen Medical Centre, Nijmegen, The Netherlands

**Keywords:** Search guide, systematic review, education and training

## Abstract

Before starting a new animal experiment, thorough analysis of previously performed experiments is essential from a scientific as well as from an ethical point of view. The method that is most suitable to carry out such a thorough analysis of the literature is a systematic review (SR). An essential first step in an SR is to search and find all potentially relevant studies. It is important to include all available evidence in an SR to minimize bias and reduce hampered interpretation of experimental outcomes. Despite the recent development of search filters to find animal studies in PubMed and EMBASE, searching for all available animal studies remains a challenge. Available guidelines from the clinical field cannot be copied directly to the situation within animal research, and although there are plenty of books and courses on searching the literature, there is no compact guide available to search and find relevant animal studies. Therefore, in order to facilitate a structured, thorough and transparent search for animal studies (in both preclinical and fundamental science), an easy-to-use, step-by-step guide was prepared and optimized using feedback from scientists in the field of animal experimentation. The step-by-step guide will assist scientists in performing a comprehensive literature search and, consequently, improve the scientific quality of the resulting review and prevent unnecessary animal use in the future.

Prior to setting up a new animal experiment, thorough analysis of previously performed experiments is paramount from both the scientific and ethical viewpoints. Through such analysis, the maximum amount of available information can be derived from previous work on the research topic and, as a result, unnecessary duplication of experiments can be prevented. Moreover, new insights that may arise from aggregating earlier work can be used to improve the design of future animal experiments. The method that is most suitable to carry out a thorough analysis of the literature is a systematic review (SR).^1^ An SR implies that a review has been prepared using a systematic approach to minimize biases and random errors, and that the components of the approach are documented in a ‘Materials and methods’ section.^2^

An essential first step in an SR is to retrieve all potentially relevant studies.^1^ This requires adequate formulation of the research question, followed by a comprehensive search strategy. A comprehensive search strategy strongly underlies the quality of any literature review.^3^ Not finding all relevant studies concerning a specific topic or omitting potentially relevant studies from a literature review may result in bias and, consequently, false or imprecise conclusions. When the omitted experiments are a random sample of all conducted experiments, only the precision of the outcome measure is decreased. However, when a particular selection of, for example, studies with non-significant or ‘negative’ results is not found and therefore not included, the SR will be biased towards a positive result. This so-called reporting bias may increase potential hazards involved in translating experimental outcomes to possible clinical benefits.^4–6^ A systematic search may reduce the effect of reporting bias, but does not completely solve the problem. Some reporting bias and more specifically publication bias will remain and should be further investigated when analysing the results. A central register of performed and planned experiments as well as publishing the negative results of a research project might further diminish the problem of publication bias.^5^

In contrast to the clinical field, where writing SRs and thus executing comprehensive search strategies are common practice, this is not the case within preclinical animal research.^7–8^ This is surprising, since animal studies are often used as the foundation for clinical trials. One of the reasons may be that this approach of formally summarizing the results of previously performed experiments is not yet well known to many biomedical researchers designing animal experiments. Moreover, available guidelines from the clinical field cannot be copied directly to the situation within animal research. In addition, searching for and finding all the available literature on animal studies is not an easy task^9^, even though attempts have been made to make the search process easier. Hooijmans *et al.*^10^ and De Vries *et al.*^11^ describe filters for PubMed and EMBASE, respectively, to ease the process of finding all animal studies in these major biomedical databases.

To facilitate a structured, thorough and transparent search to identify all relevant animal studies (in both preclinical and fundamental science) concerning a specific research question, we developed an easy-to-use, step-by-step search guide which was optimized through the feedback from scientists in the field of animal experimentation. This step-by-step guide will assist scientists in performing a comprehensive literature search and, consequently, improve the scientific quality of the resulting review and prevent unnecessary animal use in the future.

## Effective methodology and approach: the step-by-step guide

To prepare this step-by-step guide, we made use of the literature,^12–16^ the *Cochrane Handbook for Systematic Reviews of Interventions*^3^ and experts in the field of laboratory animal science. The experts in the field of laboratory animal science were asked to test the step-by-step guide for ease of use and to evaluate the clarity of the text. They reported their findings on an evaluation sheet with prespecified questions. The step-by-step guide was optimized through feedback from these experts.

The basic steps on how to design and carry out comprehensive search strategies to identify potentially relevant animal studies on a specific research question are presented in Table 1 and explained more fully in the following sections.

**Table 1:** Basic steps on how to design and carry out a comprehensive search strategy to identify potentially relevant animal studies on a specific research topic

Step	Details	Example
(1) Formulate research question	Formulate a focused research question, consisting of: (i) Intervention/exposure (ii) Disease of interest/health problem (iii) Animal/animal species/population studied (iv) Outcome measures	What are the effects of (i) omega-3 fatty acid supplementation on (iv) A*β* plaque load in (iii) animal models for (ii) Alzheimer's disease?
(2) Identify appropriate databases and sources of studies	Identify both general biomedical and topic-specific databases Select all relevant databases Check other sources, such as reference lists	PubMed/MEDLINE and EMBASE (Ovid)
(3) Transform research question into search strategy	Design and run a search strategy customized for each database Start with a database that includes a thesaurus, e.g. PubMed or EMBASE Involve an information specialist Save citations (titles/abstract) in reference software Document the applied search strategies	See Table 2 for details on PubMed search strategy
(4) Collect search results and remove duplicates	Combine saved citations of all databases into one file in reference software and remove citations that appear more than once	PubMed, *n* = 173; EMBASE, *n* = 506 Removing duplications (*n* = 139) Total number of unique citations, *n* = 450
(5) Identify potentially relevant papers	Screen title and abstract of the references and identify papers based on potential relevance	Screen PubMed, *n* = 173; EMBASE, *n* = 367

### 1. Formulate research question

A focused, well-formulated research question is a prerequisite for designing an optimal search strategy.

Since the main components of the research question determine the structure and the sequence of the searches, it is important to carefully identify these components. Within animal research, a specific research question generally contains the following components: (1) intervention/exposure; (2) disease of interest/health problem; (3) animal/animal species/population studied; and (4) outcome measures. A well-formulated research question could then be: ‘What is the effect of [*intervention/exposure*] on [*outcome measures*] in [*animal/animal species/population studied*] for [*disease of interest/health problem*]?’ Formulation of a focused research question is also important for building an efficient (sensitive) and low numbers needed to read search strategy.^12^ Parallels can be found within the clinical setting where the acronym PICO (Participants, Interventions, Comparisons and Outcomes) is often used to determine the critical aspects of a clinical question in intervention studies.^16^ Fundamental science could also benefit from this step-by-step search guide, because the search strategy remains the same, but the building blocks for the research question may be a bit different (e.g. it is dependent on the scientific field studied). Although other types of search components may be relevant when formulating a specific research question in fundamental science, the translation of the research question into a search strategy is similar.

### 2. Identify appropriate databases and other sources to search

To identify as many relevant papers as possible, searching in more than one database is recommended. An overview of health databases can be found at http://healthlinks.washington.edu/contentBrowser.jsp?ctype=1. The most frequently used biomedical databases are MEDLINE and EMBASE. Both databases are indexed and use thesaurus terms to facilitate an easy and more complete search. The indexing by MEDLINE and EMBASE refers to a description of documents using certain rules, vocabularies and keywords. The thesaurus refers to special vocabulary, where the relationships between terms are expressed in a standardized manner. MEDLINE and EMBASE each have a specific thesaurus. Several search interfaces are available which can be used for both databases, such as PubMed (for MEDLINE), EMBASE.com (for EMBASE), Ovid, DIMDI, Dialog and Datastar. PubMed is very widely used to access MEDLINE because it is freely available. PubMed comprises both MEDLINE and non-indexed biomedical citations. Examples of major databases without a thesaurus are Web of Science and Scopus. Besides general biomedical databases, subject-specific databases may also be selected. Within animal research, more than 100 databases and websites are available containing specific information on laboratory animal science.^9,17^ This information is not necessarily available in the more regularly used major biomedical databases. It may be worthwhile checking for potentially relevant papers in a few of these databases as well. Different bibliographic databases vary in type of publications included and the periods of time covered.^14^ Besides electronic bibliographic databases, other sources could also be used to identify relevant studies, such as reference lists in retrieved papers.^3^

### 3. Transform research question into search strategy

Once the research question is defined and the databases are selected, the search strategy can be prepared. Designing a search strategy for a specific database is a complex task. For building a comprehensive search strategy, one needs to be familiar with the specific search terms about the research topic and with the search engine's specific possibilities of searching. Literature searches can be difficult without a basic knowledge of the way information is organized and indexed. Basic and advanced courses in the use of major electronic bibliographic databases are often available at the institutional library. Taking such a course is strongly recommended. It is also advisable to involve an information specialist from the library for technical assistance when designing comprehensive search strategies. In Table 2, an overview of the detailed steps and an example of how to transform a research question into a search strategy in PubMed are given. These steps are more fully described and explained in Section 3.1. Section 3.2 describes how to transform a research question into a search strategy for other databases.

**Table 2:** Detailed steps to transform research question into search strategy in PubMed

Step	Details	Example
(A) Split research question into critical search components	Determine the critical search components (SC); usually this can be done by defining: SC1: Intervention/exposure SC2: Disease of interest/health problem SC3: Animal/animal species/population studied (SC4: Outcome measures)*	What are the effects of omega-3 fatty acid supplementation on A*β* plaque load in animal models for Alzheimer's disease? SC1: Omega-3 fatty acid supplementation SC2: Alzheimer's disease SC3: Laboratory models (SC4: A*β* plaque load)*
(B) Identify relevant search terms for search component 1 (SC1)	**Identify standardized subject terms**: Collect Medical Subject Heading terms (MeSH terms) in PubMed: Use a word processor to document this process Do some background reading to become familiar with terms related to the topic Identify relevant synonyms and related terms Use the PubMed thesaurus (in MeSH database) to explore terminology: broader/narrower terms, related terms, entry terms Perform a PubMed search for every single MeSH term Assess the number of results found per MeSH term to evaluate usefulness Evaluate the appropriateness of MeSH terms considering definition, context or number of results	**SC1: Omega-3 fatty acid supplementation** dietary fats, unsaturated [MeSH terms] fatty acids, omega-3 [MeSH terms] fish oils [MeSH term]
	**Identify free-text terms**: Collect free-text terms to search in title and abstract of references Use a word processor to document this process Use terminology used in papers concerning this topic Use Scopus or Google for investigating variation in terminology Use the singular and plural forms Use UK and US spelling Include relevant abbreviations and trademarks Use truncation carefully Perform a PubMed search for each free-text term by adding [tiab] Assess the results found per term from the search history Evaluate the appropriateness of used terminology (consider: context or number of results)	fish oil [tiab] fish oils [tiab] (fish [tiab] AND oils [tiab]) n-3 fatty acids [tiab] omega-3 fatty-acids [tiab] (omega-3 [tiab] AND fatty-acids [tiab]) PUFA [tiab] DHA [tiab] Etc. fatty acids [tiab]: **78,653***(too many irrelevant)* hits omega-3 fatty acids [tiab]: **2779** hits
	**Combine MeSH terms and free-text terms**: Use the Boolean operator ‘OR’ to combine both MeSH terms and free-text terms. This will result in search result: **SC1**	dietary fats, unsaturated [MeSH Terms] OR fish oils [MeSH term] OR fatty acids, omega-3 [MeSH terms] OR DHA [tiab] OR fish oil [tiab] OR fish oils [tiab] etc. **SC1: 64,783 hits**
(C) Repeat step B for SC2	This will result in search result: **SC2**	SC2: Alzheimer's disease **112,958 hits**
(D) Combine search results for SC1 and SC2	Use the Boolean operator ‘AND’ to combine search results for SC1 and SC2 from the search history. This will result in search result: **SC1 AND SC2**	SC1 AND SC2: **431 hits**
(E) Evaluate search results	Assess the number of results and the relevance of the records and, if necessary, prompt rethinking of search terms	
(F) Repeat step B for remaining components (i.e. SC3 and occasionally SC4*)	If SC3 or SC4 is to select all animal studies, a recently developed filter can be used (Hooijmans *et al.*^10^) This will result in search result: **SC3 AND SC4**	SC3: Laboratory animals **4,936,738 hits** (filter used in Hooijmans *et al.*^10^) SC4: A*β* plaque load **113,037 hits**
(G) Combine search results of all the separate SCs	Use the Boolean operator ‘AND’ to combine all the separate SCs from the search history. This will result in search result: **SC1 AND SC2 AND SC3 (AND SC4)**	SC1 AND SC2 AND SC3: **173 hits** SC1 AND SC2 AND SC3 AND SC4: **78 hits**
(H) Evaluate search results	Assess the number of results and the relevance of the records and, if necessary, prompt rethinking search terms and repeat steps B–G	
(I) Transfer search results into a reference software	Save citations (reference + abstract + source) in reference software	

*Often scientists do not include SC4 (outcome measures) in the search strategy, because many abstracts do not contain a description of these outcome measures.

By including SC4 in the search strategy, there is a potential risk of missing relevant studies

#### 3.1 Transform research question into search strategy for PubMed

##### A. Split research question into critical search components

As mentioned in step 1 of Table 1, the research question often contains four components. These components can be translated into the following search components (SCs): (SC1) intervention/exposure; (SC2) disease of interest/health problem; (SC3) animal/animal species/population studied; and (SC4) outcome measures. Because, in many papers, outcome measures are only described in the main article and are rarely indexed, including SC4 (outcome measures) in a search strategy might increase the risk of missing relevant studies. SC4 (outcome measures) should therefore not always be included in the search strategy.

##### B. Identify relevant search terms for each SC

For each of the critical SCs, a separate search string needs to be developed. Each component-specific search string includes an extensive collection of appropriate search terms. The use of appropriate terminology is the cornerstone of an effective search. Using inappropriate terms may result in missing relevant studies or in too many irrelevant studies and a high number needed to screen/read. Search terms can be divided into standardized subject terms and free-text terms.

*B1. Identify Medical Subject Headings (MeSH) terms for SC1* Standardized subject terms are terms that are assigned to papers by indexers. In MEDLINE, the standardized subject terms are called Medical Subject Headings (MeSH terms). Standardized subject terms are useful because these terms provide a way of retrieving all indexed articles that use different words to describe the same topic (e.g. when searching for papers about Alzheimer's disease (‘Alzheimer disease’ [MeSH]), also papers in which, for example the entry term ‘presenile dementia’ is used, are found).

PubMed provides the MeSH database to explore relevant terminology and synonyms and to see the relationships between the terms. This can help to find the appropriate terminology.

For identification of MeSH terms and documentation of the search strategy, it can be practical to use a word processor, such as MS Word. The potentially relevant MeSH terms, including synonyms, e.g. entry terms, can be copied to the Word file. By studying the entry terms in the Word file, new relevant MeSH terms may be identified. The finally selected MeSH terms used in the search are then documented accordingly.

MeSH terms should be checked for appropriateness. The appropriateness of a single MeSH term can be evaluated by performing a PubMed search (add [MeSH] to search term in search bar) and assessing how the retrieved results cover the subject. When, on evaluation of the search results, it turns out that too many irrelevant studies are identified, or that the search resulted in too few hits, reformulation of the search terms is necessary.

*B2. Identify free-text terms for SC1* Besides MeSH terms, free-text terms also need to be identified when designing a comprehensive search strategy. It is important to use free-text words in addition to MeSH terms because not all papers that are searched through PubMed have already been indexed. It may take 2–6 months until indexing is finished (L. Bowes, US National Library of Medicine, personal communication). Recently published papers may therefore not yet be indexed and consequently be missed. Another reason for using free-text words is that papers are not always correctly indexed.

To be complete, all possible relevant free-text terms need to be identified. It is practical to start with the MeSH terms and entry terms mentioned in the MeSH database of PubMed. Subsequently synonyms and related terms can be identified. Relevant articles (often reviews), Scopus, Google, etc. can be used for this purpose. Here, common text words may be identified as well as the index terms that the indexers have assigned to the articles. Free-text words should include all synonyms in singular and plural forms, UK and US English spelling, trade names, relevant abbreviations and trademarks. For example, when searching for ‘docosahexaenoic acid’ a frequently used abbreviation is DHA (Table 2, step B). Common spelling errors should also be covered; for example, ginko biloba for ginkgo biloba.

To economize the search string, truncation [adding * to a word stem] is often used in combination with free-text words. Truncation allows finding all the terms that begin with a given text string. For example, if you search statistic*, PubMed will simultaneously retrieve words such as statistic, statistics, statistical, etc. However, unwisely truncated terms may dramatically increase the number of unwanted results. The advice is to handle truncation with care and always check the search details for relevance.

To economize the search strategy, you can add [tiab] after a search term in PubMed and consequently your search will be restricted to the title and abstract of article citations and other less useful parts of the citation (such as author names and addresses) will not be searched. When [tw] is added after a search term, PubMed will search for that specific word in all subject-related fields in the article citation. This may result in irrelevant papers, for example, where the author's name is also the name of an intervention.

Free-text words should also be checked for appropriateness. The appropriateness of free-text terms can be evaluated by performing a PubMed search (add [tiab] or [tw] to the free-text terms in the search bar) and assess how the retrieved results cover the subject, how many hits there are and the context in the search history.

For identification of free-text terms and documentation of the search strategy, it can again be practical to use a word processor, such as MS Word. The finally selected free-text terms used in the search are documented as well.

*B3. Combine MeSH terms and free-text terms for SC1* After collecting appropriate MeSH terms and free-text words for SC1, all these terms need to be combined into one search string. This can be done by combining all search terms, MeSH terms + free-text terms, with the Boolean Operator ‘OR’. When this search string is used in PubMed, it results in search results for SC1. The search string for SC1 is saved in order to be used in future search actions and for reporting purposes.

Since the aim of the comprehensive search strategy is to retrieve as many relevant studies as possible, high sensitivity is necessary. Sensitivity, in this context, refers to a search that identifies as many research articles as possible that are potentially relevant. This may result in low precision and, consequently, in many hits. This is common to a comprehensive search strategy.

##### C. Repeat steps B1–B3 for SC2

After identifying relevant search terms for SC1, steps B1–B3 are repeated for SC2.

##### D. Combine search results for SC1 and SC2

After retrieving search results for SC1 and search results for SC2, they have to be combined into one search string in order to retrieve potentially relevant papers. Papers containing both SC1 and SC2 search results are then identified. The Boolean operator ‘AND’ can be used to combine search results for SC1 AND SC2 from the search history.

##### E. Evaluate search results

When developing a comprehensive search strategy, there needs to be a balance between comprehensiveness and precision. To be comprehensive, you need high sensitivity. As a result, the precision may be low and the number of hits may be high. This is not necessarily a problem since there is a third and maybe a fourth SC to combine the search result with to obtain more specific results and less hits.

##### F. Repeat steps B1–B3 for the remaining SCs (i.e. SC3 and occasionally SC4)

To prepare a search string for SC3 (animal/animal species/population studied) steps B1–B3 can be repeated. To identify all animal studies in PubMed, Hooijmans *et al.*^10^ designed an animal search filter, the so-called ‘Animal filter’. The ‘Animal filter’ is freely available from the publisher's website and can be copied and pasted into the search bar in PubMed. Use of this filter is easy and improves the search efficiency.

As mentioned before, not all components of the research question lend themselves well to searching. For example, if SC4 is an outcome measure that is often not well described in the title or abstract of an article and is not well indexed with MeSH terms, then this component should not be included in the search strategy to prevent missing relevant studies.

##### G. Combine search results of all the separate SCs

**Figure 1:**
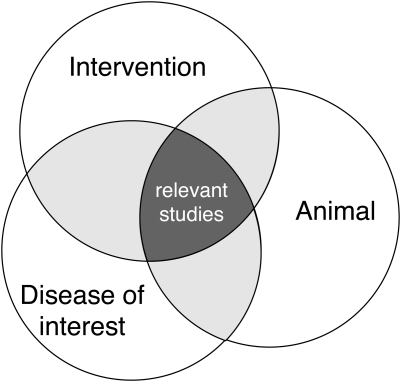
Combining components in the search strategy (adapted from Higgins and Green^3^)

In a final search, all separately developed search strings for each SC need to be combined to retrieve only the potentially relevant studies (Figure 1). This can be done by combining all the separate search results for each SC from the search history in PubMed using the Boolean operator ‘AND’. This results in final search string: SC1 AND SC2 AND SC3 (AND SC4). The final search string can be saved in a Word file and in PubMed for future use, to update the search results and to be able to report the exact search string in the review published. PubMed also has the functionality to automatically apply a specifically saved search string to most recently published or indexed articles. The reference details of the articles identified in this way will be sent to the researcher by email.

##### H. Evaluate search results

Similar to step E, the final search results of search string SC1 AND SC2 AND SC3 (AND SC4) have to be evaluated for relevance. In case the search result SC1 AND SC2 AND SC3 (AND SC4) retrieves too many hits, the terminology in the search string can be adjusted and narrower terms can be selected. In order to be as complete as possible, it is quite usual to have 3000–5000 hits. However, one should not be set back by 8000–12,000 hits at this stage. In Section 5, we describe how to deal with these numbers of hits. When a search string is too narrow, low numbers of hits will be found (e.g. less than 300). Broader terms should be selected in order to be as complete as possible in a search. Developing search strategies is an iterative process in which the terms used are modified, based on what has already been retrieved. This means that based on the number of hits and their relevance, the search terms can be adjusted to being broader or narrower.

To restrict the number of hits in the search strategy, the use of limits in PubMed may seem attractive. However, limits should be used with caution to prevent missing potentially relevant studies and consequently introducing potential bias. From clinical literature reviews, for example, it is known that to prevent selection bias, there should be no restriction on the language of the identified studies, because ‘negative results’ are often published in non-English journals.^13^ There should be a rationale for applying limits to a search strategy and it should be indicated in the text of the review.

##### I. Transfer search results into a reference manager program

The citations of the search result SC1 AND SC2 AND SC3 (AND SC4) are transferred and saved from PubMed into a reference manager program. Examples of such programs are: Endnote, Reference Manager, Procite and RefWorks. The saved citations in the reference program should at least include the reference details and the abstract.

#### 3.2 Transform the research question into a search strategy for other databases

Besides through PubMed, other databases should be used to identify as many relevant papers as possible. The search strategy for PubMed described in Section 3.1 can be (slightly) adjusted and used to design a search strategy for other databases and search engines. One should be aware that different databases use different levels of keyword systems and different levels when indexing material, if any. Again it is advisable to involve an information specialist from the library when designing comprehensive search strategies, especially when you are not experienced in searching in specific databases.

##### EMBASE

A major biomedical database that also includes standardized subject terms is EMBASE. In EMBASE, the standardized subject terms are called EMTREE terms. A similar approach to find standardized subject terms and free-text words as used in Table 2 can be applied to prepare a search strategy in EMBASE. When searching on free-text terms in the search string of EMBASE in the Ovid interface ‘.ti,ab.’ is added to each search term. To find all animal studies in EMBASE, an animal search filter was developed by De Vries *et al.*^11^

##### Other databases

Besides MEDLINE and EMBASE, other databases could be considered. As stated before, for each database, the search-engine-specific search terminology needs to be found and applied. Not all databases use standardized subject terms. If a database does not use standardized subject terms, careful selection of free-text terms is very important. In many cases, a well-developed PubMed or EMBASE search strategy can be ‘translated’ for such databases by copying the search string per SC and stripping the field indicators, such as [MeSH], [tiab] and [tw]. Examples of databases not using standardized subject terms are Web of Science and Scopus.

### 4. Collect search results of all databases and remove duplicate citations

After developing a search strategy for each database and collecting the citations from the separate databases, the search results need to be combined into one file in the reference program. Citations that appear more than once can then be removed. Duplicate citations often appear because databases can encompass the same journals. After removing the duplicate citations, a list of potentially relevant papers is created.

### 5. Identify potentially relevant papers

The list of the potentially relevant papers needs to be screened for relevance of the studies with regard to the research question. The first screening can be done very quickly using only the title and abstract of the studies. To prevent bias in the selection process, it is very useful to have two observers screening the papers independently for relevance. The criteria used for the first screening are based on the SCs. For example, when the citation clearly does not describe an animal study, is not a primary study or is not studying the disease of interest, these citations can be removed since these are not relevant to the research question. Only clearly irrelevant citations should be removed (i.e. one should be over inclusive to prevent incorrect removal at this stage).

**Figure 2:**
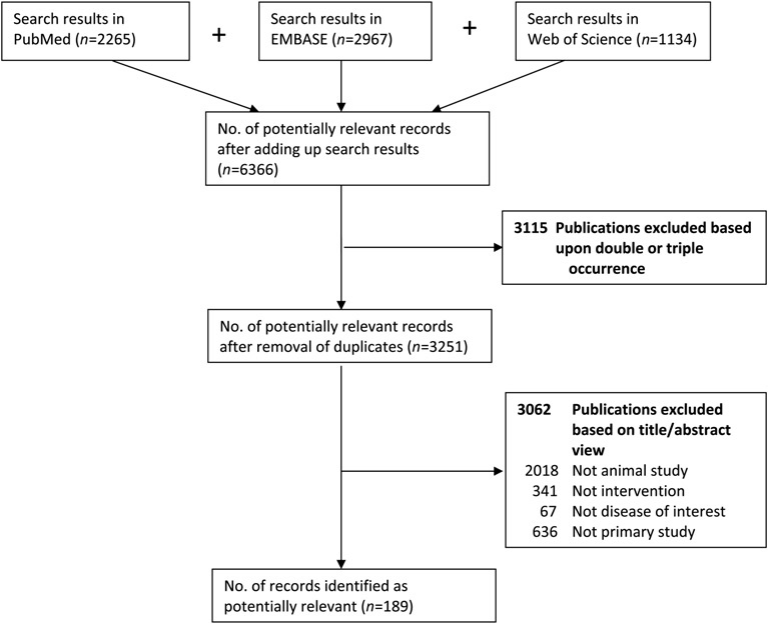
A fictive example of reporting on search results and reasons for exclusion of studies

The citations resulting from the first screening undergo a second screening when writing an SR. The second screening is based on predefined in- and exclusion criteria and is also performed by two independent reviewers. Throughout the entire screening process for potentially relevant papers the reasons for removal of citations should be documented and reported to facilitate transparency and to make it possible for an interested reader to independently examine accuracy of study removal (Figure 2).

## Documenting and reporting the search process

The search processes should be documented in enough detail to ensure correct reporting in the (systematic) review and for the reproducibility of the searches in all databases used. Detailed documenting of the search process is also important to keep track of identified studies and to be able to update the search at a later state. Reporting criteria for the search process in SRs in human research are available.^15^ In short, to be systematic, explicit and transparent, the scientist should always report: (1) all databases and other sources searched; (2) the dates of the last search for each database and the period searched; (3) full search strategies (including all search terms) for each database; and (4) any language or publication status restrictions used. An example of reporting on search results and reasons of exclusion of studies is given in Figure 2.

## Concluding remarks

A comprehensive search strategy is a key element to ensure the quality of a (systematic) review and the validity of its findings.^3^ Searching for pertinent literature on animal studies can be very difficult.^9^ To facilitate a structured, thorough and transparent search process to identify potentially relevant animal studies concerning a specific research question (in both preclinical and fundamental science), we developed an easy-to-use, step-by-step search guide. The practical guide presented in this paper is meant to assist and stimulate scientists in performing a comprehensive literature search and adequately document and report on the search process. Transparency on the search process and adequate reporting makes it possible for others reading the review to judge the thoroughness of the search, and thereby the potential of bias in the review. This practical guide cannot be used to identify unpublished studies. Leaving out unpublished studies may result in an overestimation of the effect size because unpublished studies often contain negative data.^5^ It should be noted that this step-by-step guide should be used with caution, because this guide is not exhaustive. It is highly recommended to always involve an information specialist for assistance when designing comprehensive search strategies.
